# Supplementary material for: Extracting meaningful features from time series analysis of plasma analytes in clinical research
Source: medRxiv. 2026 Oct 11:2026.10.08.26365091. Preprint. [Version 1] doi: 10.64898/2026.10.08.26365091 (PMC13692660; doi:10.64898/2026.10.08.26365091)
Supplement: Supplementary file 1 [file NIHPP2026.10.08.26365091v1-supplement-1.pdf]

medRxiv preprint doi: https://doi.org/10.64898/2026.10.08.26365091; this version posted October 11, 2026. The copyright holder for this preprint (which was not certified by peer review) is the author/funder, who has granted medRxiv a license to display the preprint in perpetuity.
This article is a US Government work. It is not subject to copyright under 17 USC 105 and is also made available for use under a CC0 license.

Additional File 1

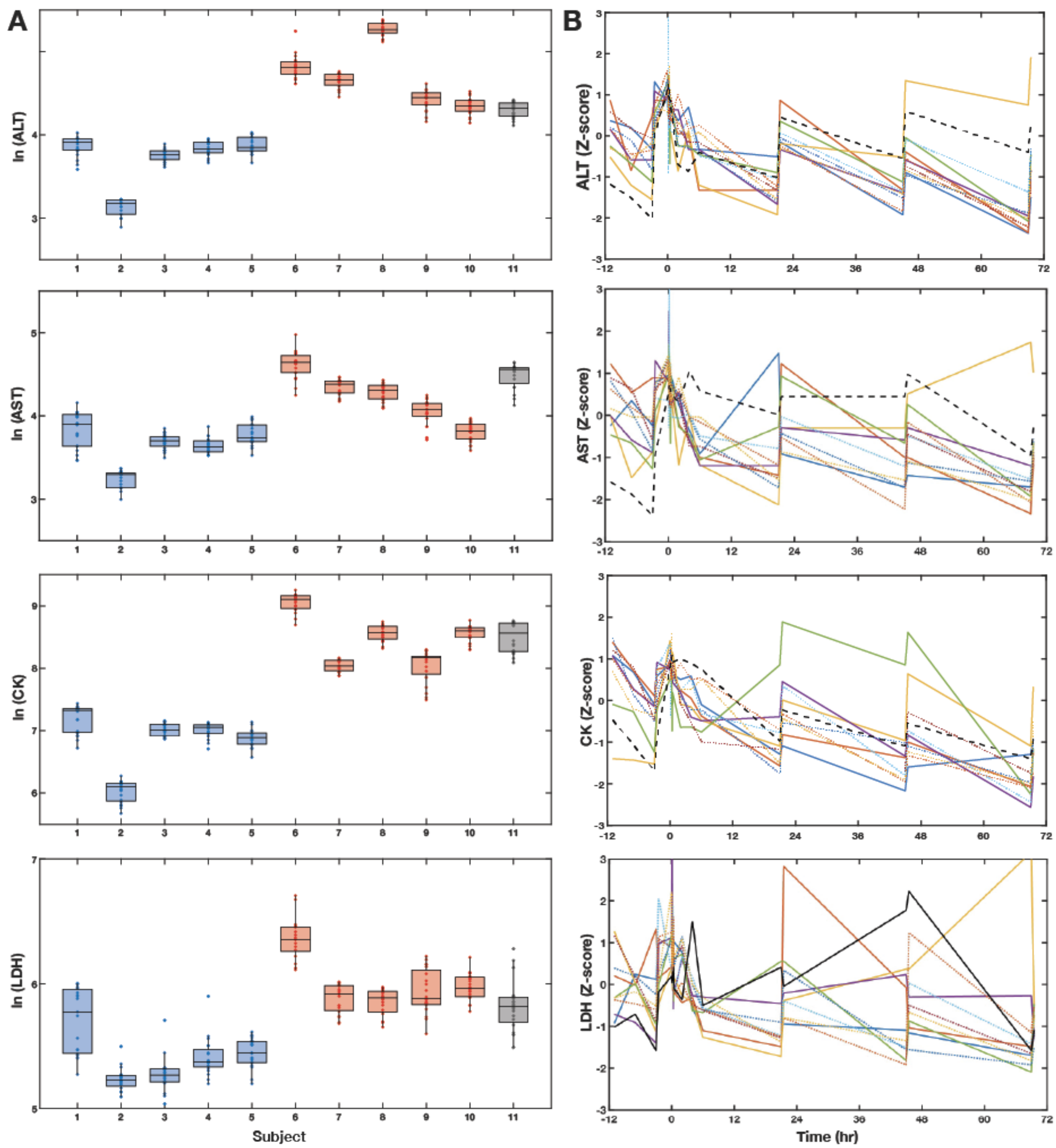

Additional Figure 1: Transformed analysis of plasma ALT, AST, CK and LDH. A) Box plots of log transformed plasma ALT, AST, CK, and LDH by participant clustered by LGMD subtype (blue- R12 Anoctamin5-related; red- R2 Dysferlin-related; black- R9 FKRP-related). B) Z-transformed plasma enzyme levels for each participant over the study time course.

medRxiv preprint doi: https://doi.org/10.64898/2026.10.08.26365091; this version posted October 11, 2026. The copyright holder for this preprint (which was not certified by peer review) is the author/funder, who has granted medRxiv a license to display the preprint in perpetuity.
This article is a US Government work. It is not subject to copyright under 17 USC 105 and is also made available for use under a CC0 license.

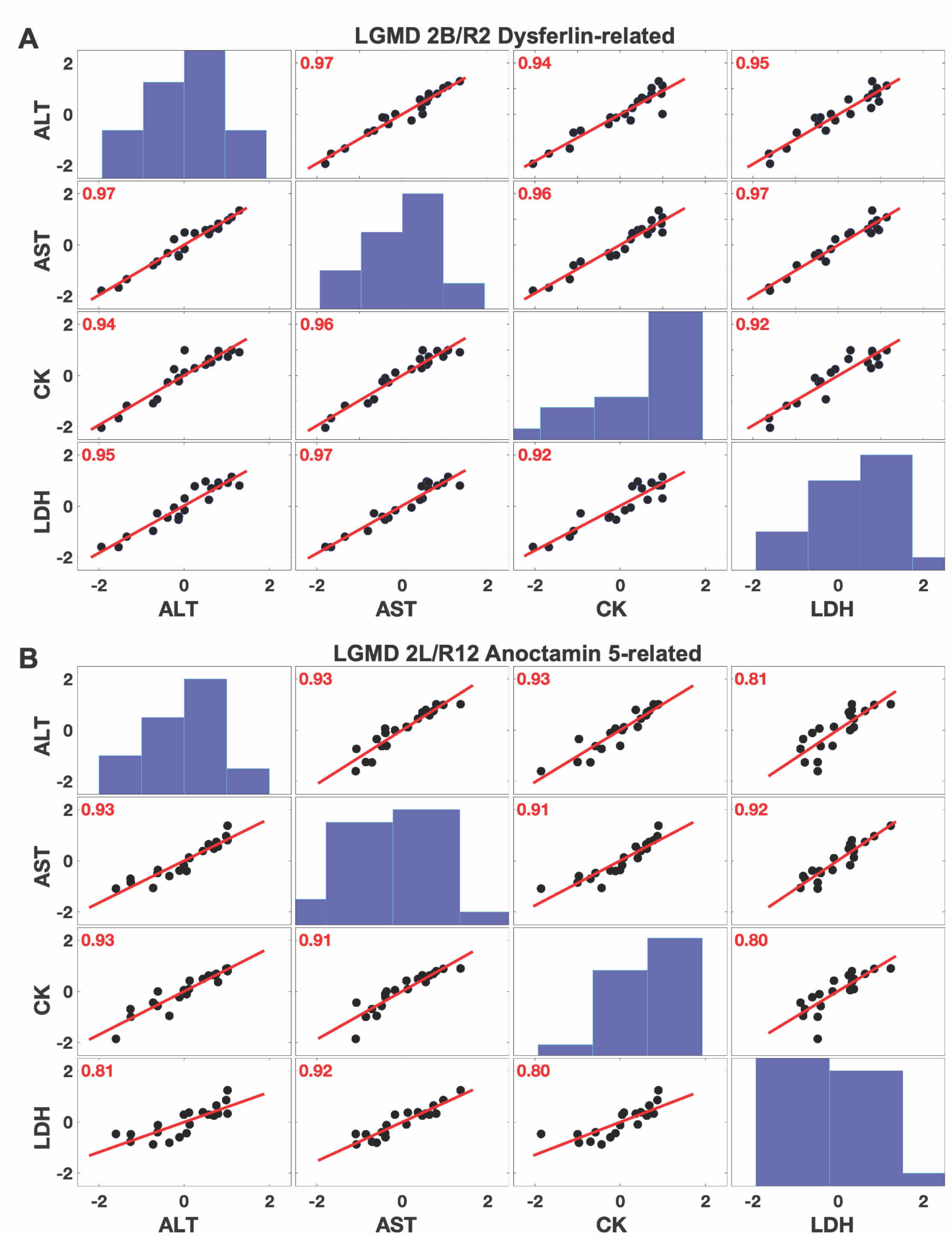

Additional Figure 2: Pearson correlation analysis of ALT, AST, CK and LDH by LGMD subtype. A) LGMD 2B/R2 Dysferlin-related; B) LGMD 2L/R12 Anoctamin 5-related. The distributions of data are represented as histograms along the diagonal. All correlations are significant at the p = 0.05 level.

medRxiv preprint doi: https://doi.org/10.64898/2026.10.08.26365091; this version posted October 11, 2026. The copyright holder for this preprint (which was not certified by peer review) is the author/funder, who has granted medRxiv a license to display the preprint in perpetuity. This article is a US Government work. It is not subject to copyright under 17 USC 105 and is also made available for use under a CC0 license.

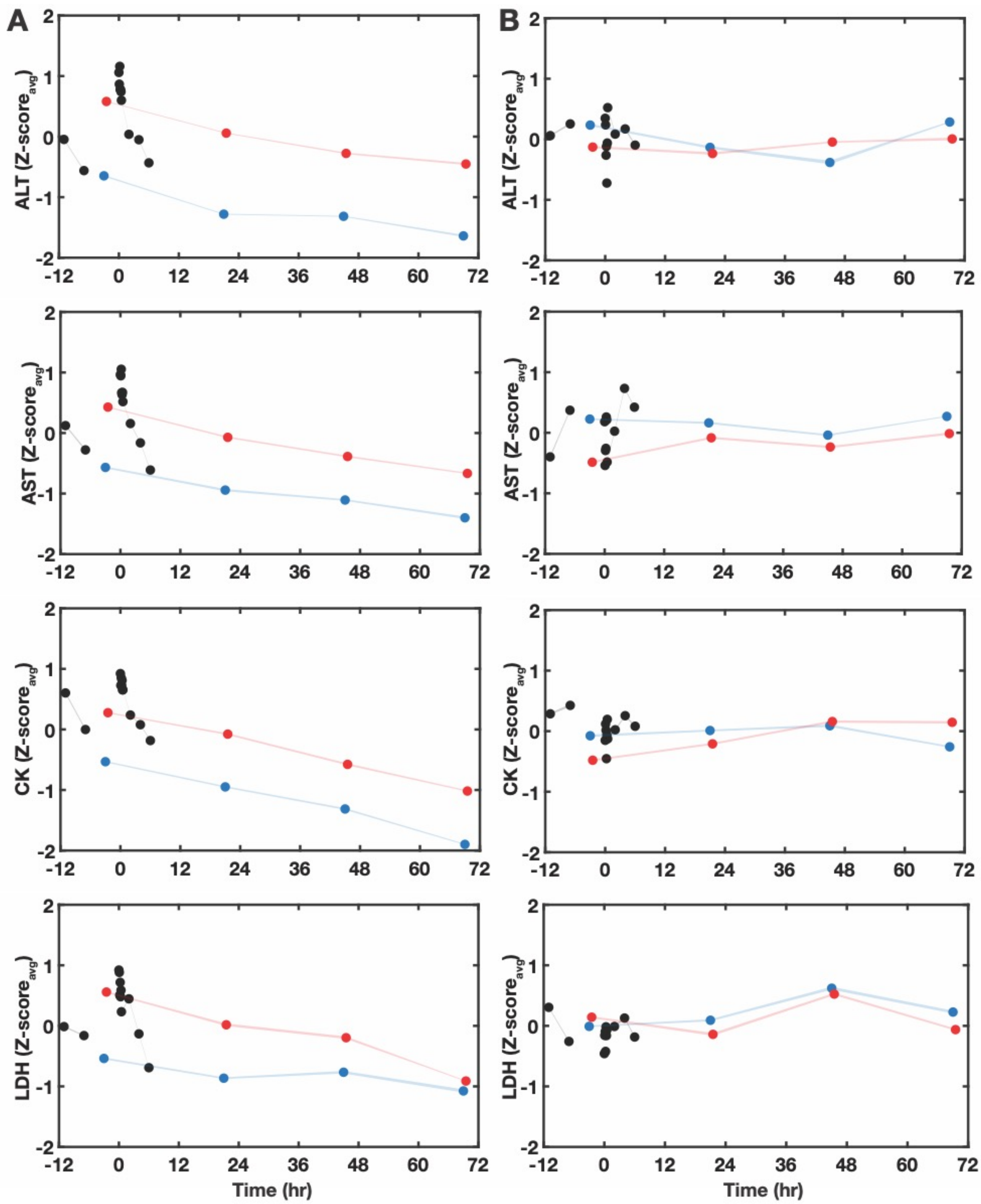

Additional Figure 3: Average Z-score of plasma enzymes with random permutations in time. Average participant plasma ALT, AST, CK, and LDH Z-scores, without (A) and with (B) random permutations in time, plotted against time (in hours) with physical assessment at time 0. Blue and red timepoints correspond to pre- and post- morning activity; black points correspond to post-arrival and post-physical assessment.

medRxiv preprint doi: https://doi.org/10.64898/2026.10.08.26365091; this version posted October 11, 2026. The copyright holder for this preprint (which was not certified by peer review) is the author/funder, who has granted medRxiv a license to display the preprint in perpetuity. This article is a US Government work. It is not subject to copyright under 17 USC 105 and is also made available for use under a CC0 license.

Additional Table 1: A simplified model was used to fit the log transformed data, with one time constant for the high-frequency data, as the full feature model used for the Z-score data did not converge when applied to the log transformed data.

Feature model	**Fitted Values (-,+ 95% CI)**			
	ALT	*AST*	*CK*	*LDH*
Tau 2 (hr)	41.82 (29.8, 53.85)	31.3 (23.44, 39.16)	34.59 (24.36, 44.82)	36.87 (18.82, 54.92)
Tau long (hr)	691.5 (398.7, 984.2)	518.6 (326.4, 710.8)	344 (253.8, 434.2)	362 (142.7, 581.2)
